# Retraction Note: Effect of liver transplants with retrograde reperfusion on early postoperative recovery of liver function and its risk factors

**DOI:** 10.1186/s12893-026-03725-2

**Published:** 2026-04-15

**Authors:** Jiajia Shen, Ming Wang, Chengkai Yang, Qiucheng Cai, Yi Jiang, Xiaojin Zhang

**Affiliations:** Department of Hepatobiliary and Pancreatic Surgery, 900th Hospital of Joint Support Force, Fuzhou city, 350025 China

**Retraction: BMC Surgery 24, 174 (2024)**

**https://doi.org/10.1186/s12893-024-02467-3**

The Editor has retracted this article. An investigation conducted after its publication discovered that it contains material that substantially overlaps with the following article [1].

The authors did not reply to correspondence from the Publisher about this retraction.
